# Implementing large-scale data quality validation in a national arthroplasty registry to improve compliance: the National Joint Registry data quality audit programme

**DOI:** 10.1302/2633-1462.39.BJO-2022-0051.R1

**Published:** 2022-09-14

**Authors:** Christopher Boulton, Carol Harrison, Timothy Wilton, Richard Armstrong, Elaine Young, Derek Pegg, J. Mark Wilkinson

**Affiliations:** 1 National Joint Registry, Health Quality Improvement Partnership, London, UK; 2 NEC Software Solutions UK, Hemel Hempstead, UK; 3 Mid Cheshire Hospitals NHS Foundation Trust, Crewe, UK; 4 Department of Oncology and Metabolism, University of Sheffield, Sorby Wing, Northern General Hospital, Sheffield, UK

**Keywords:** Joint arthroplasty, Data quality audit, Outcomes, Quality improvement, Registry, arthroplasty registries, National joint registry, cemented total hip arthroplasty, arthroplasty surgery, elbow, hips, knees, revision surgery, elbow surgery, periprosthetic joint infection

## Abstract

Data of high quality are critical for the meaningful interpretation of registry information. The National Joint Registry (NJR) was established in 2002 as the result of an unexpectedly high failure rate of a cemented total hip arthroplasty. The NJR began data collection in 2003. In this study we report on the outcomes following the establishment of a formal data quality (DQ) audit process within the NJR, within which each patient episode entry is validated against the hospital unit’s Patient Administration System and vice-versa. This process enables bidirectional validation of every NJR entry and retrospective correction of any errors in the dataset. In 2014/15 baseline average compliance was 92.6% and this increased year-on-year with repeated audit cycles to 96.0% in 2018/19, with 76.4% of units achieving > 95% compliance. Following the closure of the audit cycle, an overall compliance rate of 97.9% was achieved for the 2018/19 period. An automated system was initiated in 2018 to reduce administrative burden and to integrate the DQ process into standard workflows. Our processes and quality improvement results demonstrate that DQ may be implemented successfully at national level, while minimizing the burden on hospitals.

Cite this article: *Bone Jt Open* 2022;3(9):716–725.

## Introduction

Good clinical audit requires good quality data. The National Joint Registry (NJR),^1^ established in 2002 and covering England, Wales, Northern Ireland, the Isle of Man, and Guernsey, collects information on arthroplasty surgery for the purpose of performance monitoring and quality improvement. The key objectives of the NJR are to promote patient safety and to improve clinical standards for the benefit of patients, clinicians, and the orthopaedic sector as a whole. The NJR has established standardized minimum datasets for primary and revision hip, knee, shoulder, elbow, and ankle arthroplasty surgery to ensure that data are collected in a consistent way and that comparisons may be drawn fairly. Although the NJR captures over 200,000 arthroplasty cases each year, there is a clear impetus to ensure that the quality of the dataset is high.

In the setting of arthroplasty registries, key quality metrics include compliance (the proportion of procedures performed that are entered onto the registry and measured against independently collected administrative data); consent (the proportion of patients undergoing those procedures who have consented for their personal identifiers to be used by the registry); and linkability to outcome events (the presence of a valid set of identifiers that can be used to match the record to revision and mortality events).^2^

The aim of this article is to describe the effect of the implementation of the NJR’s formal data quality audit (DQA) programme on data compliance. We describe the development of the DQA process and how the annual cycle is operationalized. Finally, we outline the impact of the DQA on data compliance since implementation and explain the future direction of the embedded DQA programme through automation.

## NJR data quality prior to the DQA programme

**Fig. 1:**
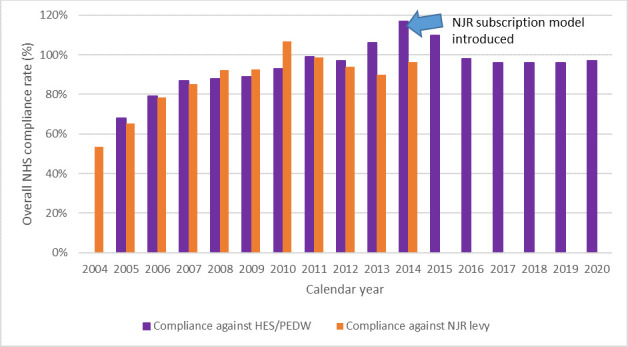
Overall NHS compliance rate by calendar year. Compliance is defined here as both a percentage of relevant procedures in Hospital Episode Statistics (HES)/Patient Episode Database for Wales (PEDW) with a corresponding NJR record, and as a percentage against NJR levy payments. The blue arrow represents the change in funding model.

From 2003, when the NJR began collecting data, it was mandatory to record hip and knee arthroplasty procedures from the independent sector. For NHS procedures, while there was very strong professional encouragement, case entry was not made mandatory by the Department of Health until April 2011. The initial funding model consisted of a levy system in which orthopaedic implant manufacturers paid a fee to the NJR for each construct they sold. This fee was added to the sale price of the implant. This system continued from 2003 to 2014, after which a subscription based model was adopted with hospitals and industry both subscribing to NJR services. The levy system generated an additional source of data from which the NJR could compare sales numbers with the corresponding records in the NJR. This gave a crude estimate of the completeness of the NJR, as not all sold prostheses were implanted. Although for the initial four years of the registry compliance was suboptimal, with under 90% of cases entered compared with the sales figures, after 2008 compliance was in excess of 90% and on occasion greater than 100%. When compliance was over 100%, this was an artefact of the practice of stockpiling prostheses (Figure 1).

Comparing procedures to a levy was not sufficiently refined to distinguish within year rates of compliance nor differences in compliance between primary and revision procedures. An alternative comparator was therefore needed, and the Hospital Episode Statistics (HES) dataset maintained by NHS Digital has been used for this purpose for English NHS organizations since 2006. The comparison of data entry on to the NJR and HES data and Patient Episode Database for Wales (PEDW) gave a clear indication of data missingness, but did not establish a mechanism for missed cases to be picked up (Figure 1). It was also unable to examine independently funded procedures. For this reason, a formal audit cycle capable of reconciling the two sources of data and allowing their correction was set up using data from each NHS organization’s Patient Administration System (PAS) and each independent sector organization’s business administration system. Table I shows the performance of the NJR against metrics of compliance, consent, and linkability.

**Table I.** Annual compliance, consent, and linkability rates for National Joint Registry (NJR) procedures from 2003 to 2020.

Operation calendar year	Procedures, n	Consent, n	Consent rate, %	National identification number recorded, n	Linkability rate, %	NHS compliance rate, %	NJR compliance rate, %*
2003	55,157	33,298	60.4	30,691	55.6	N/A	53.6
2004	102,257	64,768	63.3	60,600	59.3	68	65.2
2005	127,981	93,713	73.2	89,920	70.3	79	78.6
2006	132,976	107,005	80.5	107,143	80.6	87	85.3
2007	153,043	127,818	83.5	138,806	90.7	88	92.2
2008	161,173	140,421	87.1	153,430	95.2	89	92.8
2009	163,933	143,605	87.6	157,747	96.2	93	106.9
2010	170,014	151,933	89.4	164,356	96.7	99	98.8
2011	177,347	161,095	90.8	172,023	97.0	97	93.9
2012	191,119	174,727	91.4	185,934	97.3	106	89.9
2013	197,276	182,204	92.4	192,191	97.4	117	96.2
2014	217,505	202,663	93.2	212,177	97.6	110	N/A
2015	223,772	207,815	92.9	218,324	97.6	98	N/A
2016	234,875	216,402	92.1	229,250	97.6	96	N/A
2017	239,445	222,531	92.9	233,775	97.6	96	N/A
2018	235,680	220,306	93.5	229,477	97.4	96	N/A
2019	241,764	227,174	94.0	234,966	97.2	97	N/A
2020	124,556	112,643	90.4	118,180	94.9	89†	N/A

* Based on levy collection.

† Data entry/year end incomplete at the time of writing.

N/A, not applicable.

Patient consent, in particular, represents a crucial element of data quality (DQ) for the NJR, since this forms the basis under which confidential clinical data can be used by the registry. In cases from England where it is not clear whether a patient has consented or not, identifiable data are collected and used in the same way as those from consenting patients under our Health Research Authority Confidentiality Advisory Group ethical approval.

## Drivers for a formalized data quality strategy

Alongside NJR’s own ongoing assessment of its DQ, participant organizations are encouraged to examine their own DQ by carrying out local audits against key indicators including rates of compliance, consent, and linkability. For example, Kosy et al^3^ performed a local audit of the attribution of surgery to named surgeons and found that their unit had been allocating cases to the wrong consultants in over one-third of cases. Note that each hospital organization may have more than one joint arthroplasty operating site, here termed a “unit”. It is at the unit level upon which the data are reported to the NJR. Patients may be admitted or assessed under one surgeon and operated upon by another, affecting interpretation of an individual surgeon’s performance but not that of the unit or the implants involved. A broader exercise comparing NJR submissions with explanted prostheses from the London Implant Retrieval Centre found a systematic under-reporting of revision surgery to the NJR, with almost 40% of the revisions in the study being absent from the NJR.^4^ It should, however, be noted that implants sent to retrieval centres represent a small and highly selected group that is not representative of overall national revision practice. The NJR DQA demonstrates that baseline revision compliance has been consistently above 85% since 2014. We also build on work conducted by international registries in Australia,^5^ New Zealand,^6^ and Sweden,^7^ which examined the accuracy of reporting of revision surgery for periprosthetic joint infection and found missingness of between 25% and 33%.

By 2012 it was apparent that data completeness in the NJR had reached ~95% when compared to routinely collected administrative data, but was no longer appearing to improve. It was felt that the overall accuracy and completeness could be improved by cross-referencing both the NJR and HES/PEDW data against each unit’s PAS to confirm the individual missing cases in each dataset. It was also recognized that this does not, of itself, correct and improve the case ascertainment. The need for a feedback loop for actively acquiring the missing cases and entering them in retrospect was thus apparent, but no published audits appeared to demonstrate such a system in action.

## Development and principles of NJR data quality strategy

**Fig. 2:**
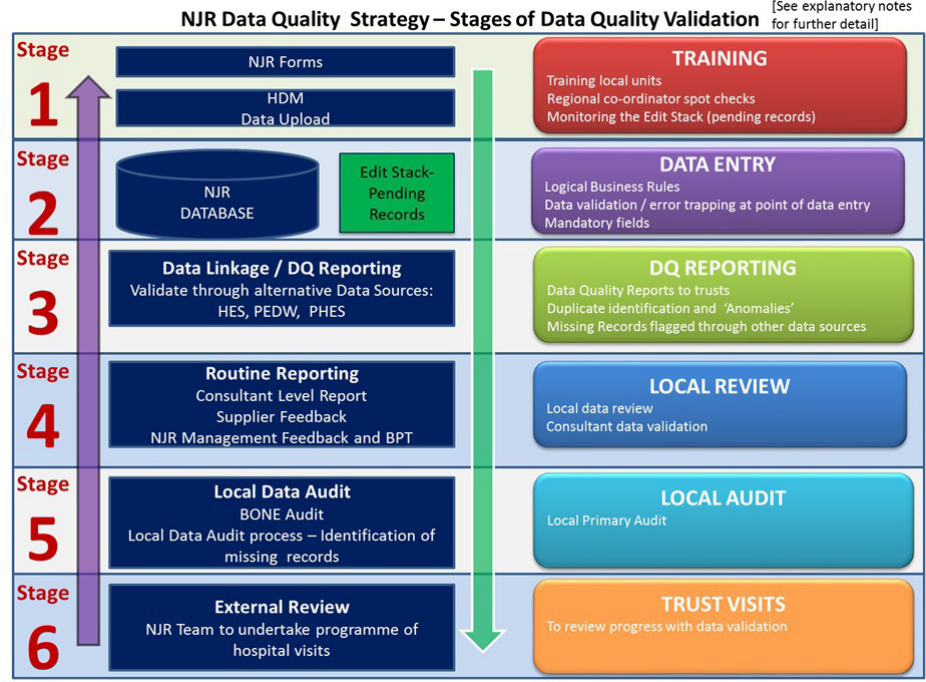
Overview of the National Joint Registry’s (NJR) data quality strategy. HDM, Health Data Management; DQ, data quality; HES, Hospital Episode Statistics; PEDW, Patient Episode Database for Wales; PHES, Private Hospital Episode Statistics; BPT, Best Practice Tariff.

A DQ strategy committee was established with the support of NHS England, patient representatives, and other key stakeholders. This group comprised orthopaedic surgeons, patient representatives, specialists in data from the NJR’s statistical analysis and data management contractors and members of the NJR management team. The remit of the group was to lead the development and delivery of the NJR’s DQ strategy and to oversee the roll-out of the NJR DQA programme. An outline of the strategy is shown in Figure 2, with explanatory notes in Supplementary Tables i and ii.

All organizations contributing to the NJR were sent an annual statement. This represented cases found on the registry but missing from that unit’s PAS data, along with a similar list of those cases found in the PAS data but not in the NJR data. In this paper-based system, data entry managers at each organization were asked to check the individual unit records and upload the correct data to NJR. A system of data completeness awards was developed to give an incentive for having good systems to capture NJR data. NHS purchasers and providers also agreed target levels for NJR data completeness that were required to obtain top-up payments for their joint arthroplasty activity.

Once the process was rolled-out to all NHS units, rapid returns were forthcoming from some units but others required more encouragement and support. At the same time, it was decided that a complete audit of recent years’ data would be more valuable than a less complete audit capturing the start of the NJR, when data were more incomplete and involved a greater number of legacy implants.

## Operationalizing the NJR data quality strategy

During finalization of the DQ strategy, six units conducted a pilot of using a proposed NJR toolkit (Supplementary Material). From February 2015, each unit worked with the NJR to share data and understand areas for improvement. Three brief case studies then focused on the issues identified that included: the identification of records within the unit PAS system, but not entered on the NJR; NJR forms having been found in patients’ notes, but not entered onto the NJR; the incorrect coding of procedures; the incorrect recording of surgery dates; and incorrect consultant in charge identity. Similar findings have persisted across the years of audit, and these themes have represented much of the focus and consequent improvement year on year.

Following review of the outcomes of the pilot and approval of the final strategy, work commenced on finalizing the processes required to conduct the first formal audit. A data quality lead was established at each unit. Communications included raising awareness of the importance of the audit through NJR sub-committees, regional events, e-bulletins, newsletters, local Clinical Audit Meetings, and through the use of British Orthopaedic Association communication channels.

Process and procedure documents were agreed for each step of the audit process undertaken by the NJR team and the unit-nominated DQ lead. The audit tool piloted earlier was finalized as a means to: 1) semi-automate the process of validating returned audit data; and 2) provide a mechanism to track every stage of the audit for each organization by recording progress metrics against individual unit records. This enabled clear audit status reporting back to the NJR DQ committee. Alongside this, a compliance audit report was developed to be sent to the chief executive officer (CEO) and clinical lead for each organization containing the key findings, recommendations, and additional learning points from the audit process. The report was intended to provide each organization with their own key learning points to act upon.

**Fig. 3:**
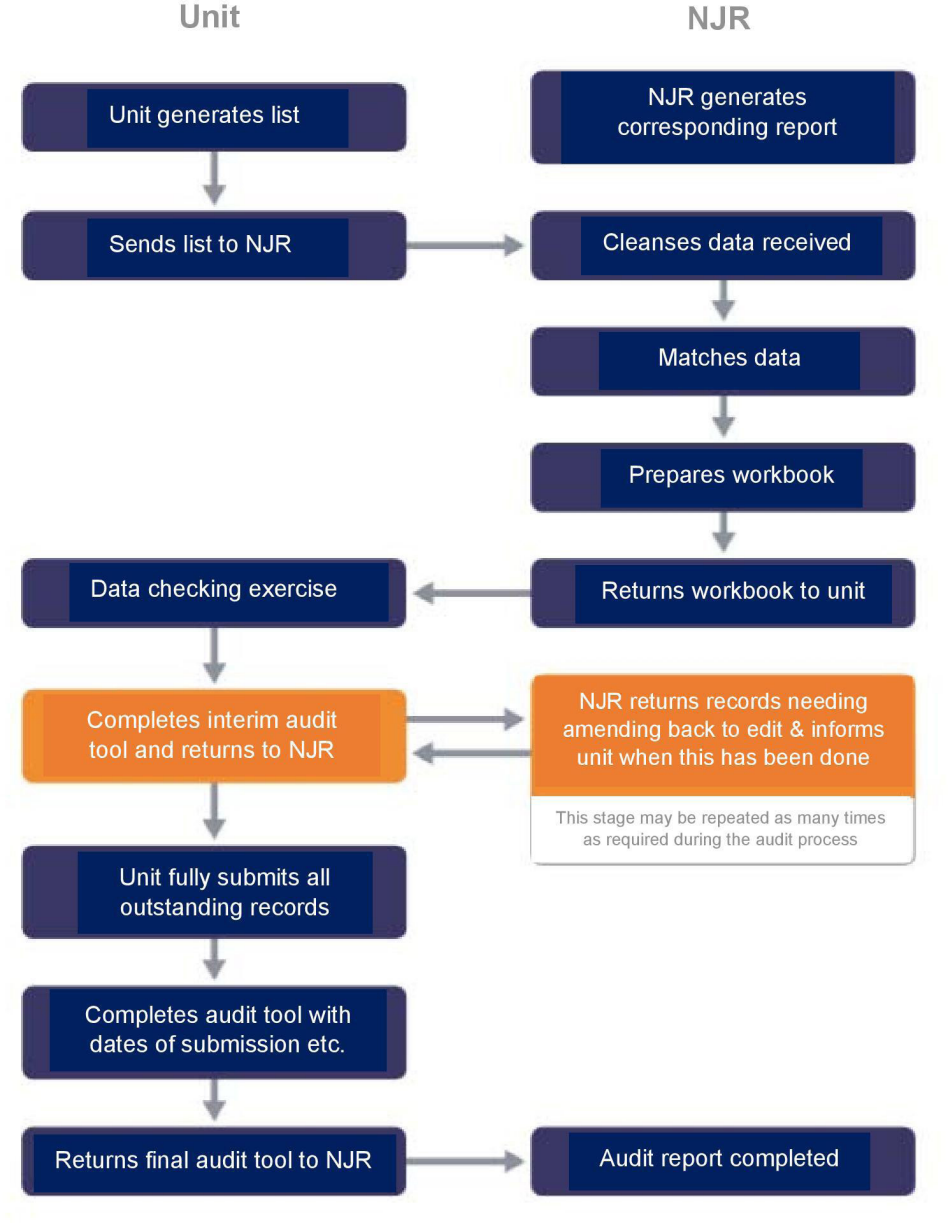
Process flow for National Joint Registry (NJR) data quality audit.

The first audit year was 1 April 2014 to 31 March 2015 (FY2014/15), and focused on NHS organizations. Participating organizations were asked to send data from their local PAS systems to the NJR for the specified audit period, identified using the appropriate Office of Population Censuses and Surveys Classification of Interventions and Procedures (OPCS-4) codes.^8^ These data items consisted of a local unit patient identifier, date of operation, procedure type, and consultant in charge. The NJR team generated a corresponding report from the NJR data entry system, and the validation process shown in Figure 3 was then followed.

An in-depth review of the outcomes and process was undertaken following completion of the first audit, and improvements in the process were identified and implemented. Through this process it was determined that: 1) the audit should be completed at the unit level, rather than at organizational level; and 2) that a key DQ contact should be identified for each unit so that a direct relationship between that contact and the NJR could be developed. Included in this strategy was the selective undertaking of individual unit visits by senior members of the DQ strategy group to support those that were struggling to achieve the data entry target. Alongside this, a need to enhance documentation was identified and a unit data template was thus established. Further refinement of user guides based on feedback was also required, as was enhancement of the information provided within the final audit report to include an audit action plan and in later reports providing year-on-year unit achievement figures to support good practice.

During October and November 2016 the second NJR DQA commenced. This cycle was conducted at unit level and further extended to include the independent sector. In total 412 units participated in this audit. After each DQA year was completed, and as the units fully engaged in completing the audits and provided feedback, the process was reviewed.

## Results for each audit cycle

**Fig. 4:**
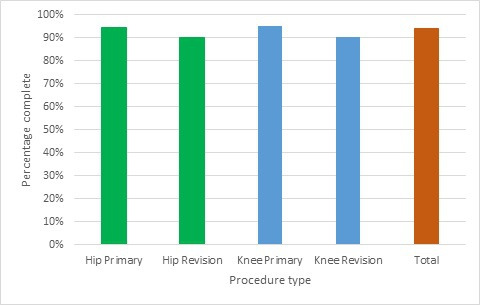
Summary of completeness for 2014/15 data quality audit.

FY14/15 was carried out at the NHS organization level. Figure 4 shows the outcome of the first audit year, and demonstrates the baseline completeness prior to any missed cases being entered. An average organization-level compliance figure of 92.64% prior to any intervention demonstrated good capture in many places, but there remained over 7,000 cases where a record was present in the provider data without a corresponding record in the NJR. There appeared to be no difference in the capture rates between hip and knee surgery. There was a higher percentage of revision cases that were missed, with 9.7% of revision hips missing versus 5.6% of primaries and 9.8% revision knees versus 5.1% of primaries.

**Fig. 5:**
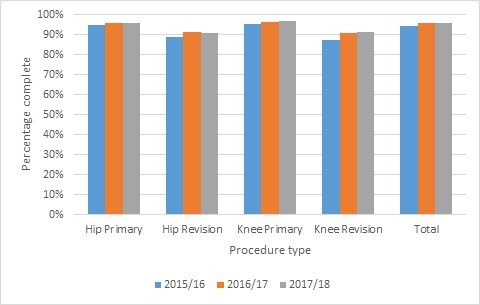
Summary of completeness for 2015/16 to 2017/18 data quality audit.

From 2015, the audit was conducted at the individual unit level and included the independent sector units. This demonstrated an average baseline unit level compliance rate of 91.78% in 2015/16, increasing to 92.46% in 2016/17 and to 94.12% in 2017/18. Figure 5 describes completeness by procedure type with a year-on-year improvement across all types, but with a persistent gap between primary and revision procedures. The overall number of missing cases reduced from 11,285 in 2015/16 to 8,670 in 2017/18.

**Fig. 6:**
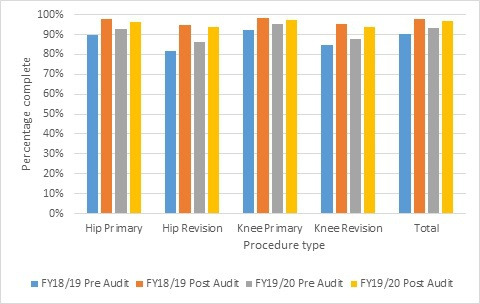
Effect of the data quality audit on completeness for 2018/19 and 2019/20.

Baseline performance of data completeness shows a year-on-year improvement across all units. For the 2018/19 audit year – the first year of automation – 61.5% of units achieved 95% or higher compliance upon first run of the audit, consistent with the 63.1% achieved in the previous year. This increased to 76.4% of units for the 2019/20 period. Figure 6 shows the pre- and post-audit cycle percentage completeness by joint and primary versus revision. It should be noted that the year 2019/20 audit is currently ongoing and has been impacted by changes in staffing related to the COVID-19 pandemic, as well as a direct instruction from NHS England for units to pause data entry for national clinical audits and registries.

## Limitations of the process and ongoing development

While the key metrics of compliance, consent, and linkability give a good overall sense of registry DQ, the nature of collecting data from over 400 units nationwide means that inconsistency of reporting and varying degrees of clinical oversight will be reflected in the data entered.

A particular area that has become a focus in recent years has been the correct entry of a complete set of component data for each procedure. NJR’s annual report now reports on a ‘whole construct’ basis, meaning that an incomplete set of components entered for a case would be classed as ‘unconfirmed’ and excluded from some analyses. Work to examine these unconfirmed components has commenced across key areas. These include elbow surgery, reverse shoulder arthroplasty, dual mobility hip arthroplasty, and multiple-unicompartmental knee arthroplasty. This ongoing work is led by the NJR DQ committee and the relevant specialist societies, and will involve both validation of component classification with industry and examination of individual cases by units. In the case of elbow surgery, a nationwide DQA led by the British Elbow and Shoulder Society (BESS) has recruited surgical trainees to examine the operative notes of cases that are either absent from the registry (but present in administrative data) or with unconfirmed constructs, in order to improve the completeness and accuracy of recorded elbow procedures.

Poor levels of response from some organizations to the NJR DQ programme have made completion of each audit a challenging and lengthy process. It became clear that the level of resources assigned to the DQA programme by both NJR and by each unit to fulfil the DQA needed to be reduced over time. The NJR determined that the effort associated with the DQA should become part of the ‘business as usual’ NJR process, as DQ becomes embedded into local processes rather than being a one-off annual task.

## Automating the process

**Fig. 7:**
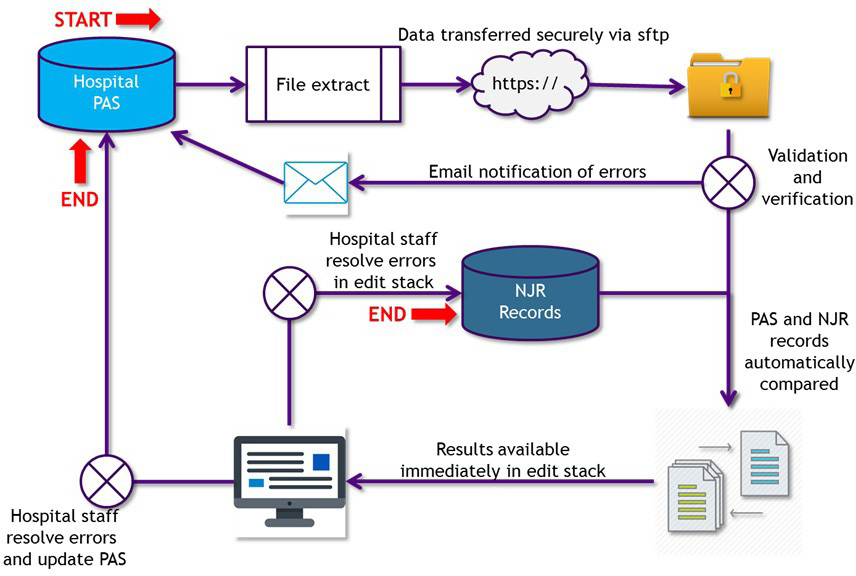
Process flow diagram for automated data quality audit. NJR, National Joint Registry; PAS, Patient Administration System; sftp, secure file transfer protocol.

In order to address the burdens identified above, in 2020/21 NJR began a national roll-out of a semi-automated audit process, as shown in Figure 7. An automated DQ platform was developed to allow the upload of PAS data direct to the NJR data entry system. It also allowed users to upload PAS data at their convenience and to produce real-time reports of compliance of NJR submissions. For example, users may upload their data monthly in arrears during the year to better manage the workload. Following reprocessing, an updated compliance percentage is displayed providing motivation for users to correct all missing or incorrect entries so that the 95%+ target can be achieved.

This process greatly reduces the number of potential missing cases that have to be checked each time the audit is run, and also allows the unit to receive their Quality Data Provider certificate in real time as the unit achieves the required criteria. A new reporting suite also supports the programme by providing comprehensive information on the status of each unit in the audit cycle, and exception reports flag any areas of concern for the NJR Compliance team to manage locally with the unit. The automated audit process is now part of units’ ‘business as usual’ process and allows them to take responsibility locally to ensure target compliance is achieved and maintained.

Positive results from the early pilot were supportive of this method rapidly becoming part of the normal workflow. This roll-out is underway for hip, knee, elbow, ankle, and shoulder data and 314 units have now completed an audit of their 2018/19 data, with 199 units having completed an audit of 2019/20 data and 46 units having started auditing their 2020/21 data. Full roll-out of the automated system was implemented at the end of 2020/21.

## Take home messages

Implementation of a DQ process has had a substantial effect on maintaining the high quality of data in the NJR, with data now routinely above 95% complete at baseline, increasing to up to 97% nationally after completion of the audit. Furthermore, many units are routinely capturing 100% of cases within each cycle.

Being able to establish and maintain this level of DQ requires investment. The effort required to support units in engaging with the audit process should not be underestimated. We have mitigated this to some extent by implementing new technology to partially automate the process, but a degree of direct support is still required.he establishment of DQA as ‘business as usual’ has meant that NJR has been able to increasingly focus on more targeted examination of areas of DQ concern relating to implants.

Other registries looking to implement similar processes would be encouraged to consider the following points: 1) a data quality committee should be established to set the strategic aims and oversee and monitor the process; 2) clear communications should be targeted to named DQ leads at each unit involved with the registry; 3) registry staff should be appropriately resourced to support units with engaging with the process, particularly in the early years of implementation; and 4) where possible, technological solutions should be implemented to reduce the burden on units and help establish these processes as part of routine registry participation.

In conclusion, DQ of clinical audits and registries is critical to ensure that derived inferences about the healthcare system being evaluated are valid. The NJR has developed a comprehensive programme of DQAs that allow individual units to use local administrative data to identify cases that have been missed by their NJR data collection systems. Units are then able to enter retrospectively data for missed cases, maximizing the completeness of the NJR and its ability to monitor outcomes. This process has not only improved overall compliance by capturing missed cases, but has improved the quality of data collection systems overall, meaning that baseline compliance figures at first run of the DQA are increasing year on year. The DQA system is now routinely embedded in unit workflows and operates on a semi-automated basis, reducing burden for unit teams and allowing more frequent auditing to take place as required. Finally, the COVID-19 pandemic has had a substantial negative impact on the UK’s current ability to provide arthroplasty surgery.^9^ Accurate real-time audit data can provide a timely and informative view on activity trends to help empower recovery plans that support appropriate resource allocation.

**Take home message**

- The National Joint Registry (NJR) gathers clinical data to inform best practice in large joint arthroplasty.

- In this paper we describe critical steps in the evolution of our good quality data audit programme that matches every NJR record with its corresponding hospital record.

- The described process provides a template for good quality data audit.
